# Clinical and epidemiological features of paracoccidioidomycosis due to *Paracoccidioides lutzii*

**DOI:** 10.1371/journal.pntd.0007437

**Published:** 2019-06-04

**Authors:** Rosane Christine Hahn, Anderson Messias Rodrigues, Paula Portella Della Terra, Andréia Ferreira Nery, Hugo Dias Hoffmann-Santos, Hellen Meira Góis, Cor Jesus Fernandes Fontes, Zoilo Pires de Camargo

**Affiliations:** 1 Federal University of Mato Grosso, Faculty of Medicine, Laboratory of Mycology/Research, Cuiabá, Mato Grosso, Brazil; 2 Federal University of Mato Grosso, Júlio Muller University Hospital, Mato Grosso, Brazil; 3 Federal University of São Paulo, Paulista School of Medicine, Department of Microbiology, Immunology and Parasitology, Cellular Biology Division, São Paulo, São Paulo, Brazil; Universidad de Antioquia, COLOMBIA

## Abstract

**Background:**

The fungus *Paracoccidioides lutzii* was recently included as a new causative species of paracoccidioidomycosis (PCM) and most cases have been reported from Brazil. According to available epidemiological information, *P*. *lutzii* is concentrated in the Middle-West region in Brazil, mainly in the state of Mato Grosso. However, clinical and laboratorial data available on patients infected with *P*. *lutzii* remain extremely limited.

**Methodology/Main findings:**

This work describes the clinical manifestations of 34 patients suffering from PCM caused by *P*. *lutzii*, treated along 5 years (2011–2017) at a reference service center for systemic mycoses in Mato Grosso, Brazil. Adult rural workers (men), aged between 28 and 67 predominated. All patients had the chronic form of the disease, and the oral mucosa (n = 19; 55.9%), lymph nodes (n = 23; 67.7%), skin (n = 16; 47.1%) and lung (n = 28; 82.4%) were the most affected sites. Alcohol intake (n = 19; 55.9%) and smoking (n = 29; 85.3%) were frequent habits among the patients. No patient suffered from any other life-threatening disease, such as tuberculosis, cancer or other inflammatory or infectious parasitic diseases. The positivity in culture examination (97.1%) was higher than that found for the direct mycological examination (88.2%). Particularly, one patient presented fungemia at diagnosis, which lead to his death. The time elapsed between the initial symptoms and the initiation of treatment of PCM caused by *P*. *lutzii* was 19.7 (31.5) months, with most patients diagnosed 7 months after the symptoms’ onset.

**Conclusions/Significance:**

Compared with the classical clinical-epidemiological profile of PCM caused by *P*. *brasiliensis*, the results of this descriptive study did not show significant clinical or epidemiological differences that could be attributed to the species *P*. *lutzii*. Future studies may confirm or refute the existence of clinical differences between the two fungal species.

## Author summary

Paracoccidioidomycosis (PCM) is an endemic mycosis in Latin America with high incidence in Brazil. The fungi *Paracoccidioides brasiliensis* (including genetic groups S1, PS2, PS3 and PS4) and *Paracoccidioides lutzii* are the etiological agents, but little is known about the clinical manifestations of PCM caused by *P*. *lutzii*. Regarding eco-epidemiological aspects, the habitat is believed to be the soil due to the predominance of the disease among rural workers and other individuals who work in contact with the land. *Paracoccidioides* spp. has been isolated from aerosol samples, armadillos and dog food, but more data are needed to better understand the ecology of this fungus. The Middle-West region of Brazil presents the highest number of cases of *P*. *lutzii* infection. It is important to note that this species presents particularities regarding the serological diagnosis in patients. Thus, this study aims to verify possible clinical-epidemiological differences in 34 patients from this geographical region. Our results do not point out significant clinical or epidemiological differences between the two species causing PCM. In Brazil, the Ministry of Health has made an effort to include this disease in the list of compulsory notification diseases in order to implement a health policy aimed at an early detection, diagnosis and treatment.

## Introduction

Paracoccidioidomycosis (PCM) is the most prevalent deep mycosis in Latin America, being endemic only in Brazil, Colombia and Venezuela. In Brazil, the state of Mato Grosso (Middle-West region), has a large number of cases, and the recently described new species *P*. *lutzii* [1] was recovered from the clinical isolates of patients from this geographic location. *Paracoccidioides lutzii* and *P*. *brasiliensis* are thermal dimorphic fungi, which grow at room temperature as mycelia and as yeasts with bipolar or multipolar buds at a temperature of 35 to 37°C (parasitic form). Estimates of annual incidence in Brazil vary from 0.71 cases to 3.70 cases per 100 thousand inhabitants [2]. According to information from the Ministry of Health, 3,181 cases of PCM deaths were recorded in Brazil between 1980 and 1995, resulting in a PCM mortality rate of 1.45 cases per million inhabitants (2.59 for the Southern region, 2.35 for the Central-West region, 1.81 for the Southeast, 1.08 for the North, and 0.20 for the Northeast) [3]. In Brazil, PCM is the 8th cause of mortality among the parasitic infectious diseases. Even so, it is still included in the group of neglected diseases, and there is no requirement for compulsory notification despite the severity of the disease and the fact that it is considered a public health problem [4]. The incidence of hospital admissions for PCM in Brazil is 7.99/1000 inhabitants, surpassing other endemic mycosis such as histoplasmosis and coccidioidomycosis [5].

The state of Mato Grosso is known as the country's granary, being the largest producer of soy, corn, cotton together with cattle breeding. This productivity is achieved due to the intense modernization of farming techniques. For this reason, most of the cases of patients affected by PCM are directly related to the agricultural activities carried out in rural properties of different territorial extensions. On the other hand, agricultural machine operators also constitute a target audience for PCM acquisition.

Recently, 65 isolates of *P*. *brasiliensis* were analyzed through nuclear and mitochondrial DNA, as well as the morphology of conidia and yeasts; in this study, the authors propose a new classification for the *P*. *brasiliensis complex* and the taxonomic recognition of the four genetic groups as *P*. *brasiliensis* (S1), *P*. *americana* (PS2) *P*. *restrepiensis* (PS3), *P*. *venezuelensis* (PS4), suggesting that they be considered as distinct species [6].

Humans and the nine-banded armadillo (*Dasypus novemcinctus*) are the accidental hosts of *Paracoccidioides* spp. and are usually infected in rural and peri-urban environments. Despite the consensus that the fungus’ habitat is the soil, few studies were able to demonstrate the isolation from this micro niche, existing many gaps concerning the knowledge on the still unresolved eco-epidemiology of PCM. Recently, *P*. *brasiliensis* and *P*. *lutzii* were detected in soil samples from three different locations in Brazil using molecular methods [7]; nevertheless little is known about the pathogenicity, virulence of strains, and more detailed aspects relating to the eco-epidemiology of the new species *P*. *lutzii*. In 2018, Hrycyk *et al*. [8] confirmed that while armadillos are highly infected by *P*. *brasiliensis*, including multiple infections by distinct genotypes or species (*P*. *brasiliensis* and *P*. *americana*) in the same animal, the same does not hold true for *P*. *lutzii*, which in turn seems to present less capacity for mycelial growth and conidial production, when developing in a soil-related condition, but this deserves further investigation.

Respiratory infection occurs via inhalation of conidia present in nature, which later reach the pulmonary alveoli. Usually, the infection is controlled by the cellular immune response, but scars can remain with latency of yeast cells. Thus, there is usually asymptomatic infection or nonspecific symptoms, or even some individuals showing the progression of infection to disease [4].

When the disease develops, the classical clinical forms are known as acute or subacute ("juvenile"), prevalent in children and young adults, in which there is inadequate Th2 cell type response to control the fungal infection. The chronic form represents 80 to 95% of the cases, affects individuals in the productive age (after the third decade of life), usually affecting the lungs, upper region including lesions in the oral mucosa, nasal mucosa, skin in places adjacent to the mouth and nose, and cervical lymph nodes. The incubation period of the disease is uncertain and may develop after many years after the individual's initial contact with the fungus [2, 4].

*Paracoccidioides brasiliensis* is composed of a cluster of molecular siblings recognized as S1 (S1a and S1b), PS2, PS3, and PS4 [9, 10]. The phylogenetic species S1a and S1b are widespread and predominantly found in lower South America, especially in the southeast and South of Brazil, Argentina, and Paraguay [10]. PS2 has a sporadic distribution and has been less frequently reported, with human cases only being reported thus far in Venezuela and the southeast of Brazil. The PS3 and PS4 species are, to date, exclusively endemic to Colombia and Venezuela, respectively [11].

Phylogenetic analyses demonstrated that *P*. *lutzii* represents a highly divergent lineage monophyletically separated from *P*. *brasiliensis*. *Paracoccidioides lutzii* is often found in the Middle-West region [1] and North [12] of Brazil, and most of the genetically evaluated clinical isolates were from the state of Mato Grosso. Regarding morphology, conidia of *P*. *lutzii* are elongated (2–22 μm), while that of *P*. *brasiliensis* measure from 2 up to 5 μm [13, 14]. To date, the main difference related to *P*. *brasiliensis* and *P*. *lutzii* lies in the serological diagnosis, where there is a need to employ local antigenic preparations in serological techniques such as ELISA, immunodiffusion and latex [15, 16].

The taxonomic description of a new species has raised the curiosity of physicians due to the possible clinical implications. Furthermore, characteristics of the *in vivo* susceptibility of *P*. *lutzii* to drugs conventionally used in the history of PCM also raise the interest of the professionals that manage patients affected by PCM.

The objective of this work was to describe the first results concerning the epidemiological and clinical characteristics of patients affected by *P*. *lutzii* from the Middle-West (Mato Grosso) of Brazil and reflect on possible similarities or differences between these characteristics and the classical profile of the disease caused by *P*. *brasiliensis*.

## Methods

### Ethical approval

This study was submitted to and approved (CAAE: 17177613.6.0000.5541) by the Federal University of Mato Grosso (UFMT) and protocol number 1796–10 by the Federal University of São Paulo (UNIFESP). All adult subjects provided informed written consent and the study was approved by ethical committee under number 288.250/CEP/HUJM/UFMT.

### Study scheme and fungal strains

**Fig 1:**
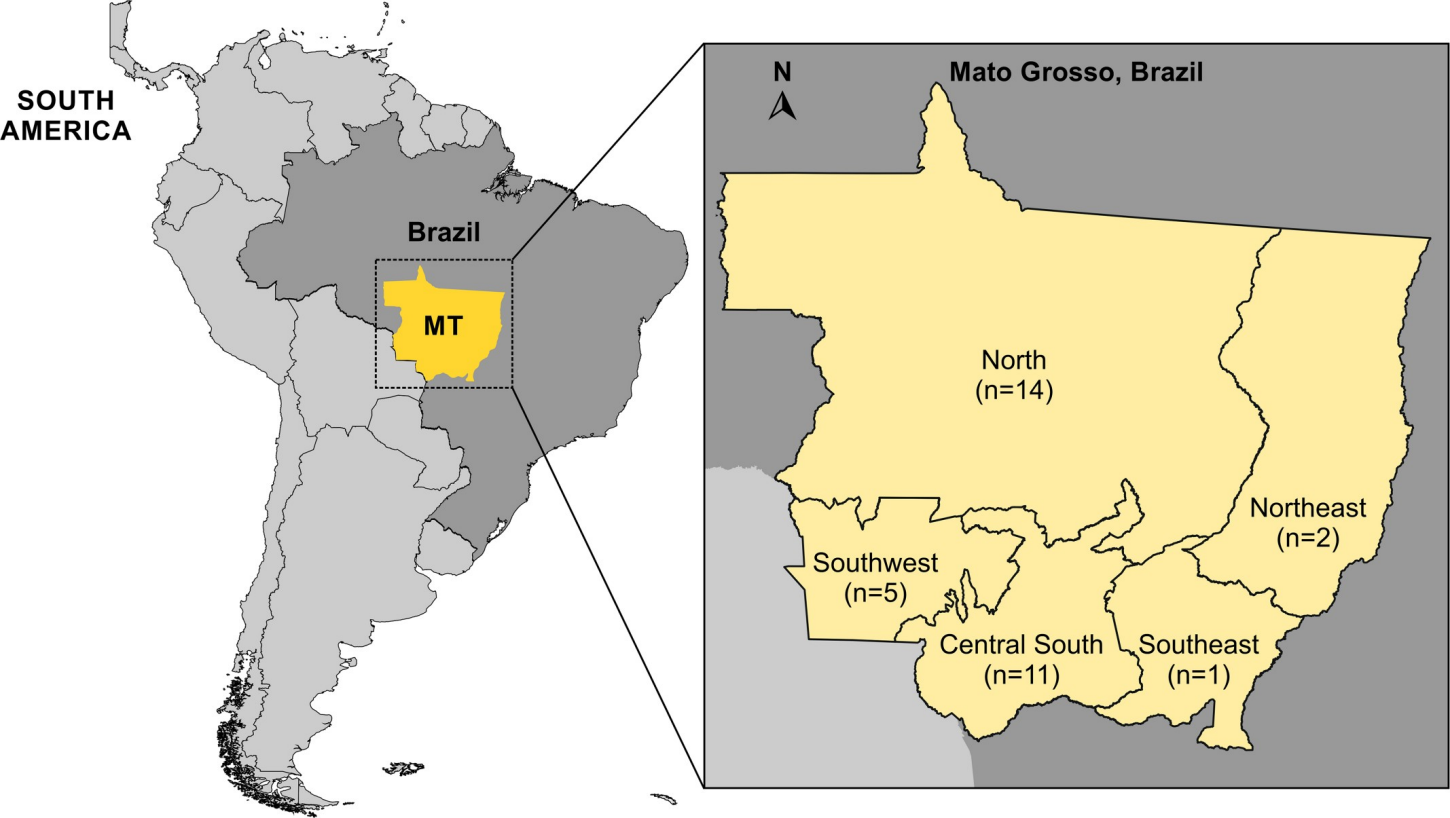
South America map showing sampling localities in Middle-West Brazil and total number of clinical cases of paracoccidioidomycosis caused by *Paracoccidioides lutzii* assessed in Mato Grosso, Brazil. The South America map was treated using the vector graphics editor Corel Draw X8.

A descriptive study was carried out on 34 confirmed PCM cases caused by *P*. *lutzii* (Fig 1), that is, those with compatible clinical manifestations and positive fungal culture for *Paracoccidioides* spp. from different clinical materials and which were later confirmed by genotyping as *P*. *lutzii*. The patients in the study were enrolled at a reference service center of systemic mycoses of the Júlio Muller University Hospital–Federal University of Mato Grosso (UFMT / HUJM), Cuiabá, Mato Grosso—Central-West region of Brazil.

The *Paracoccidioides* spp. isolates were obtained from various clinical material (sputum, cervical lymph aspiration, blood, oral mucosa scraping, scraping of the larynx, scraping of the nasal mucosa, fragment of skin biopsy). The clinical materials were cultivated in Sabouraud Dextrose Agar (DIFCO) and incubated at a temperature of 35º C in a BOD incubator (Eletrolab) for a period of up to 20 days. Colonies with cerebriform appearance and creamy color, typical of the yeast forms, were isolated with subsequent confirmation of micromorphological characteristics of *Paracoccidioides* spp.

### Genotyping of *P*. *lutzii* isolates

Isolates morphologically identified as *Paracoccidioides* spp. were subjected to molecular characterization using either *HSP70* amplification [1] or via *TUB1*-RFLP [17]. DNA was extracted and purified from fungal colonies with the Fast DNA kit protocol (MP Biomedicals). The primer pair HSPMMT1 (5’-AAC CAA CCC CCT CTG TCT TG-3’) and PLMMT1 (5’-GAA ATG GGT GGC AGT ATG GG-3’) targeting an exclusive indel region of *P*. *lutzii* were used for PCR [1]. Isolates Pb01 and B339 were used as controls of *P*. *lutzii* (positive) and *P*. *brasiliensis* (negative) respectively. In addition, for *TUB1*-RFLP, the protocol described by Roberto *et al*. [17] was used. TUB1 fragments were amplified using the primer pair α-TubF (5′-CTG GGA GGT ATG ATA ACA CTG C-3′) and α-TubR (5′-CGT CGG GCT ATT CAG ATT TAA G-3′) [18] following a double digestion with Bcl*I* and *Msp*I restriction endonucleases. The reaction contained 13 μL H_2_O, 3 μL *TUB1-*PCR product, 2 μL 10× fast digest buffer, and 1 μL each of the Bcl*I* (10 U/μL; Thermo Scientific) and *Msp*I (10 U/μL; Thermo Scientific) restriction endonucleases. The digestion mixture was incubated at 37°C for 2 hours. The digested products were electrophoresed on 2.5% (w/v) agarose gels for 120 min at 100V in the presence of GelRedTM (Biotium, USA). We included a lane loaded with 50bp DNA Step Ladder (Promega, USA). Molecular identification was performed at the Medical and Molecular Mycology Laboratory (UNIFESP/EPM). The bands generated by PCR or *TUB1*-RFLP were visualized using the L-Pix Touch (Loccus Biotecnologia, São Paulo, Brazil) imaging system under UV illumination.

### Clinical data and statistical analysis

Epidemiological and clinical data were collected from medical records of *P*. *lutzii* PCM treated patients between 2011 and 2017. The categorical variables were summarized by percentages and 95% confidence interval, and the numeric variables by mean and standard deviations. All analyses were performed by Stata Statistical Software version 12.0 (College Station, Texas, USA).

## Results

Altogether 34 patients with confirmed diagnosis of PCM were evaluated, 33 men (n = 33; 97.1%) and only one woman (2.9%), with a mean (SD) age of 46.7 (9.3) years of age. Most of the patients (75.7%) resided in the north and central regions of the state of Mato Grosso (Fig 1); 73.5% in rural areas and 26.5% in urban areas. The occupations of farmer (53.6%) and rural truck driver (32.1%) were the most frequent. Smoking (85.3%) and alcohol intake (55.9%) were very frequent among patients (Table 1). None of them suffered from other life-threatening diseases.

**Table 1:** Demographic and behavioral characteristics of patients with paracoccidioidomycosis caused by *P*. *lutzii*.

Characteristic		n	%
**Sex**	*Male*	33	97.1
	*Female*	1	2.9
**Age (years)**	*27–50*	21	61.8
	*> 50*	13	38.2
	Mean (SD): 46.7 (9.3)		
**Region of origin in MT**	*North*	14	42.4
**(n = 33)**	*Central South*	11	33.3
	*Southwest*	5	15.2
	*Northeast*	2	6.1
	*Southeast*	1	3.0
**Residence Area**	*Rural*	25	73.5
	*Urban*	9	26.5
**Occupation** **(n = 28)**	*Farming*	15	53.6
	*Rural Transport*	9	32.1
	*Other*	4	14.3
**Smoking**	*Yes*	29	85.3
	*No*	5	14.7
**Alcohol intake**	*Yes*	19	55.9
	*No*	5	44.1

**Fig 2:**
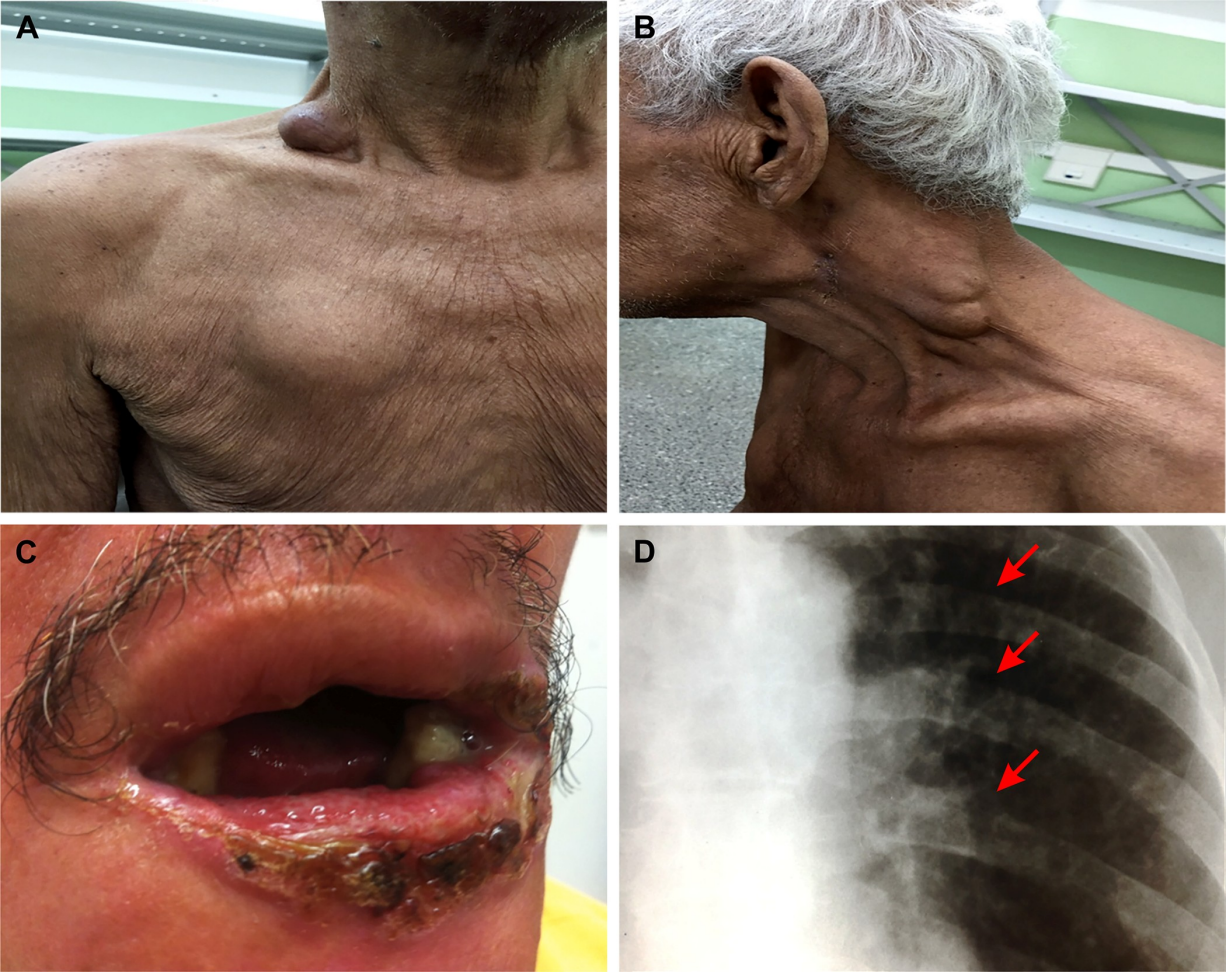
Clinical features of paracoccidioidomycosis due to *Paracoccidioides lutzii*. (A) Ganglion in the second right intercostal space on the anterior wall of the thorax in a patient with PCM caused by *Paracoccidioides lutzii*; patient 22. (B) Left cervical ganglion in a patient with paracoccidioidomycosis caused by *P*. *lutzii*; patient 09. (C) Oral lesion by *P*. *lutzii* in a patient with paracoccidioidomycosis; patient 06. (D) Bone fracture (arrow) caused by *P*. *lutzii*; patient 09.

The species *P*. *lutzii* was identified by *TUB1*-RFLP in all 34 patients described, 30 (88.2%) being new cases and 4 (11.8%) relapsed cases of the disease. The PCM in this series of cases was multifocal in 88.2% (n = 30) and unifocal in 11.8% (n = 4). All patients had the chronic clinical form of the disease, with pulmonary involvement in 82.4%, lymph nodes (Fig 2A and 2B) in 67.7%, oral (Fig 2C) in 55.9%, cutaneous in 47.1%, laryngeal in 32.8%, nasal in 11.8%, bone (Fig 2D) in 11.8% and 2.9% in adrenal glands. One of these patients had symptoms of fungemia by *P*. *lutzii*. No patient presented central nervous system or genital involvement. The average time (SD) elapsed between the initial symptoms and the initiation of treatment of PCM by *P*. *lutzii* was 19.7 (31.5) months, with most patients diagnosed 7 months after the symptoms’ onset (Table 2).

**Table 2:** Clinical and laboratory characteristics of patients with paracoccidioidomycosis caused by *P*. *lutzii* at the time of diagnosis.

Characteristic		n	%
**Admission**	*New Case*	30	88.2
	*Recurrence*	4	11.8
**Clinical form**	*Chronic*	34	100.0
	*Other*	0	-
**Classification**	*Multifocal*	30	88.2
	*Unifocal*	4	11.8
**Affected Sites**	*Lung *	28	82.4
	*Lymph nodes*	23	67.7
	*Mouth*	19	55.9
	*Skin*	16	47.1
	*Larynx*	11	32.8
	*Nose*	4	11.8
	*Bone*	4	11.8
	*Adrenal glands*	1	2.9
**Duration of symptoms**	*1–6 *	13	39.4
**(months)**	*7–12*	8	24.2
	*> 12*	12	36.4
**Diagnostic method**	*Culture*	33	97.1
	*Direct mycological examination*	30	88.2
	*Histopathological examination*	12	35.3
**Clinical Specimen**	*Lymph node aspiration*	14	41.2
	*Oral lesion scraping*	13	38.2
	*Sputum*	3	8.8
	*Skin fragment*	3	8.8
	*Blood*	1	3.0
**Drugs used for the treatment**	*SMX+TMP*	30	88.2
	*SMX+TMP and itraconazole*	7	20.5
	*Itraconazole*	2	5.9
	*Amphotericin B*	2	5.9
		**Mean (SD)**	
**Hematology/Biochemistry**	*Hemoglobin (g/dL)*	11.7 (2.6)	
	*Hematocrit (%)*	36.6 (7.1)	
	*Leukocytes (/mm3)*	10,284 (3,947)	
	*Eosinophils (/mm3)*	544 (500)	
	*Albumin (g/dL)*	3.2 (0.6)	
	*Glucose (g/dL)*	90.9 (21.1)	

SD: standard deviation

SMX+TMP: sulfamethoxazole + trimethoprim

The diagnosis of PCM was initially confirmed by culture in 97.1% (n = 33) of cases, direct mycological examination (DME) in 88.2% (n = 30), histopathological examination in 35.3% (n = 12). Clinical specimens used for the mycological exams were ganglionic secretion (n = 14), scraped oral mucosa lesion (n = 13), sputum samples (n = 3), skin biopsy (n = 3) and blood (n = 1) (Table 1).

The decision on the treatment of patients was based on the II Brazilian Consensus of Paracoccidioidomycosis [4], using sulfamethoxazole + trimethoprim in 88.2% of the patients. Out of these, 7 (23.3%) also used itraconazole and another 2 (6.9%) amphotericin B deoxycholate. The initial hematological and biochemical evaluation of the patients did not present any relevant changes (Table 2).

## Discussion

In the present study on 34 patients with confirmed infection by *P*. *lutzii*, there were no clinical or epidemiological differences that could be attributed to the *P*. *lutzii* species.

The epidemiological profile of PCM has been revealing remarkable changes in frequency, demographic characteristics and geographical distribution. More than a decade ago studies published by our research group showed differences between isolates of *Paracoccidioides* spp. Initial investigations were conducted looking for correlations between clinical forms of the disease, geographical origin of same, susceptibility to antifungal drugs and epidemiological findings [19, 20]. In 2009, Batista *et al*. [21], showed significant differences in serological test results using double radial immunodiffusion for diagnosis of PCM when sera from patients from the Middle-West and Southeast regions of Brazil were evaluated. The exoantigens obtained from isolates from patients from these geographical regions affected by PCM presented strong evidence of antigenic variation among the isolates [15, 22]. It was also observed through the RAPD technique that clinical isolates from different anatomical sites (arm and face) of a same patient presented genetic differences [23]. All evidence collected related to possible antigenic differences whenever exoantigens from different geographic locations [21] were used by different researchers who obtained results from the use of different molecular techniques seeking correlation with virulence of isolates [24] and clinical forms of the disease [20], was important for the proposal of a new species: *P*. *lutzii* [1].

However, the vast literature related to clinical, demographic and epidemiological aspects of *P*. *brasiliensis* as a single etiologic agent of the disease until 2009, highlights classical presentations fairly known by medical professionals. For the physician, it is important to assess the epidemiological, clinical, diagnostic and therapeutic impact on the disease of different species of *Paracoccidioides*, i.e., whether there are indeed differences regarding clinical manifestations between the two species: *P*. *lutzii* and *P*. *brasiliensis*, possibly attributed to the antigenic differences of clinical isolates [15], or even to the virulence of the strains [24, 25].

Taking into account the acute/subacute forms according to Ferreira [26], a multisystemic involvement of the disease is observed; the presence of lymphadenomegaly, cutaneous lesions, hepatosplenomegaly or abdominal masses. Jaundice, ascites, and peripheral edema may also be present. The latter justify the investigation of hypoalbuminemia. Signs of adrenal involvement, as well as neurological involvement, are rare in this clinical form. Digestive complaints, such as abdominal pain, chronic malabsorptive diarrhea and vomiting, are also quite frequent. Fever and weight loss complete the clinical picture, presence of growth or pain in the bone region requires the identification of bone lesions. According to Mendes [27] and Valle *et al*. [28], the chronic form is assessed through signs and symptoms related to the pulmonary, tegumentary and laryngeal involvement (cough, dyspnea, mucopurulent expectoration, ulcerated skin lesions and nasopharyngeal mucosa, odynophagia, dysphagia and dysphonia); lymphatic (adenomegaly); adrenal [29, 30] (asthenia, weight loss, hypotension, darkening of skin, abdominal pain). Relating to the central nervous system, according to Pereira *et al*. [31] and Almeida *et al*. [32] the following may be observed: headache, motor deficit, convulsive syndrome, changes in behavior and/or level of consciousness. Regarding the digestive impairment, diarrhea and sometimes malabsorption syndrome are reported [33]. In this study, all patients evaluated presented the chronic form of the disease, where pathognomonic signs and symptoms of this form were recognized, mainly showing pulmonary, lymphatic, oral and cutaneous impairment. There was no clinical evidence in this sample of patients evaluated (n = 34) that could be highlighted, considering the etiology of PCM caused by *P*. *lutzii*. One case of fungemia was observed [34], but it is not possible to infer that *P*. *lutzii* is more virulent than *P*. *brasiliensis* because of this finding. Moreover, out of the 34 cases evaluated with etiology of PCM by *P*. *lutzii*, only two were classified as severe chronic form, the majority (n = 32) being classified as moderate clinical form.

Considering the proposed species (*P*. *brasiliensis* S1a, S1b, PS2, PS3, PS4 and *P*. *lutzii*), Macedo *et al*. [35] described an autochthonous clinical case in the southeast of Brazil (Rio de Janeiro), classified as *P*. *brasiliensis* PS2. These authors reported that few cases with this molecular taxonomy have been recorded in the literature when compared with S1 and PS3, and that among the cases registered pointing PS2 as the etiologic agent a higher frequency of the chronic form of the disease was observed. This finding was also observed in 34 patients affected by PCM caused by *P*. *lutzii* assessed in this study. Associated habits (smoking and drinking) were also frequent, as well as the frequency in male individuals in the productive age. These characteristics coincide with those described in the literature for classical PCM caused by *P*. *brasiliensis* (smoking (>20 cigarettes/day for >20 years) and alcohol intake (>50g/day). They are also often associated with the mycosis) [36]. Regarding the duration of symptoms in months for patients affected by *P*. *lutzii* in this series of cases, two groups were the most frequent: 13 patients allocated in the range of 1 to 6 months, and 12 patients ranged higher than 12 months, corroborating the classical data already published for *P*. *brasiliensis*.

In terms of distribution by regions in the state of Mato Grosso, the North (n = 14) and Central South (n = 11) regions were responsible for the largest number of cases. The concentration of the highest number of cases in the Northern region can be explained by environmental factors due to the opening of new agricultural frontiers with forest felling, especially in the Amazon—Mato Grosso region [37]. In addition, the occurrence of different species of *Paracoccidioides* may also be contributing to the change in the epidemiological pattern [37].

The suspected diagnosis of PCM occurs through clinical and epidemiological data, but the confirmation is done primarily by the identification of the etiologic agent in fresh tissue examinations, cultures and histopathologic preparations, which are considered the gold standard in the definition of the disease, being known as direct techniques in the diagnosis of PCM. Indirect techniques are represented by the presence of antibodies and circulating antigens in the serum of patients with PCM. A very interesting result was found in this study, with higher positivity for the culture identification (97.1%) when compared with that found by direct mycological examination (88.2%).

Generally speaking, it is not possible, so far, to establish important clinical differences that can be attributed to *P*. *lutzii* or *P*. *brasiliensis* complex. In 2017, our research group evaluated, in another study, a total of 554 patients who were treated at the same hospital during the study period (1998 to 2014), 527 had confirmed PCM diagnosis. Out of 527 patients, 244 (46.3%) patients (mean age, 48.4 [10.9] years; range, 14–83 years), classified as the chronic form of PCM. All patients were living in rural areas, and most performed activities related to agriculture [38]. These data show that the acute form of PCM is less frequent in the state of Mato Grosso, central region of Brazil, a geographical region where a higher frequency of *P*. *lutzii* has been observed so far.

This is the first study that presents a series of cases of *P*. *lutzii*, identified by molecular methods and correlating them with the clinical and epidemiological profile of affected patients. The actual incidence of each phylogenetic species and its involvement in clinical practice should include other studies in different regions of Brazil and Latin America to compare the forms of PCM and clinical manifestations with the genetic profile of these entities.

Only a few studies are found to date in the literature offering the molecular identification of clinical isolates and their association with clinical characteristics of patients affected by PCM. For comparison purposes, considering clinical findings and molecular characterization, Macedo *et al*. [39] recently carried out phylogenetic analysis of 54 *Paracoccidioides* spp. clinical strains from Rio de Janeiro, Brazil where *P*. *brasiliensis* (n = 48) and *P*. *americana* (n = 6) were identified as the causative agents of PCM. Considering the clinical classification, the authors reported that 41 strains were identified as *P*. *brasiliensis*, 23 corresponded to the chronic form, and 16 were acute. In Mato Grosso, all 34 clinical cases infected by *P*. *lutzii* corresponded to the chronic form of PCM. In relation to the affected organs, for both *P*. *lutzii* (Table 2) and *P*. *brasiliensis* [39], lungs and lymph nodes were the most affected. Regarding the severity of the disease, 7 were classified as mild, 18 (moderate), and 16 (severe) in the case of *P*. *brasiliensis* [39], in contrast to 32 cases (moderate form) PCM caused by *P*. *lutzii*, and only 2 cases were classified as severe.

The number of clinical cases evaluated by Macedo *et* al [39] concerning *P*. *americana* is very small. Thus, it is difficult to make any inference or comparison considering clinical manifestations. This has proven to be a limitation for the study [39].

Based on the clinical findings regarding *P*. *lutzii* and *P*. *brasiliensis* complex, there is no evidence that allows us to point out significant clinical differences between species. For this reason, we believe that studies with a greater number of isolates should be conducted to confirm or refute the hypothesis that there are clinical differences related to the different species. However, the genetic susceptibility of the host should always be an important parameter to be considered, as well as the virulence of the strain, regardless of the species that causes PCM.
